# May Spasticity-Related Unpleasant Sensations Interfere with Daily Activities in People with Stroke and Traumatic Brain Injury? Secondary Analysis from the CORTOX Study

**DOI:** 10.3390/jcm13061720

**Published:** 2024-03-16

**Authors:** Salvatore Facciorusso, Stefania Spina, Alessandro Picelli, Alessio Baricich, Franco Molteni, Andrea Santamato

**Affiliations:** 1 Department of Medical and Surgical Specialties and Dentistry, University of Campania “Luigi Vanvitelli”, 80138 Naples, Italy; s.facciorusso89@gmail.com; 2 Spasticity and Movement Disorders “ReSTaRt”, Unit Physical Medicine and Rehabilitation Section, Department of Medical and Surgical Sciences, University of Foggia, 71122 Foggia, Italy; andrea.santamato@unifg.it; 3 Department of Neurosciences, Biomedicine and Movement Sciences, University of Verona, 37100 Verona, Italy; 4 Physical Medicine and Rehabilitation, Department of Health Sciences, Università del Piemonte Orientale, 28100 Novara, Italy; 5 Villa Beretta Rehabilitation Center, Valduce Hospital Como, 23845 Costa Masnaga, Italy

**Keywords:** spasticity, ICF, unpleasant sensations, stroke, traumatic brain injury

## Abstract

**Background/Objectives:** This study examined the impact of spasticity-related unpleasant sensations (pain, heaviness, stiffness) on various domains of the International Classification of Functioning, Disability, and Health (ICF) and psychosocial well-being in individuals affected by stroke or traumatic brain injury (TBI). The primary aim is to explore how these sensations affect daily activities, participation, and overall quality of life, guided by the comprehensive framework of the ICF. **Methods:** Utilizing a secondary analysis of data from a cohort undergoing Botulinum toxin type-A treatment for spasticity post-stroke or TBI, we developed and administered an ad-hoc questionnaire focusing on ICF domains such as body function, activities and participation, and psychosocial aspects such as mood, relationship, social life, motivation, and sleep quality. Spearman rho correlation was applied to assess the relationship between unpleasant sensations and functional as well as psychosocial outcomes among 151 participants. **Results:** This study identified significant correlations between the severity of unpleasant sensations and limitations in daily functioning, particularly in activities of daily living and mobility. Furthermore, an impactful association was identified between increased unpleasant sensations and deterioration in psychosocial well-being, notably in mood and sleep quality. **Conclusions:** These findings advocate for a person-centered approach in spasticity management, emphasizing the integration of sensory impairment strategies into rehabilitation to enhance functional outcomes and quality of life. Such an approach aims to improve functional outcomes and enhance the quality of life for individuals experiencing spasticity post-stroke or TBI. Future directions include targeted interventions to alleviate these sensations, support better rehabilitation results and improve patient experiences.

## 1. Introduction

Spasticity is a positive symptom of a complex condition called Upper Motor Neuron Syndrome (UMNS) [1] that affects up to 30% of patients with Stroke or Traumatic Brain Injury (TBI) [2,3]. Stroke and TBI are classified as subtypes of acquired brain injury, which encompasses brain damage incurred post-birth due to traumatic or non-traumatic causes [4]. TBI occurs due to an external impact on the head, making it part of the traumatic category. On the other hand, stroke (ischemic or hemorrhagic) is classified as non-traumatic, stemming from a disruption in blood flow to the brain, which consequently damages brain cells [5]. Spasticity could be troublesome for many patients, causing modifications in all domains of activities and participation [6]. Many therapeutic approaches provide significant benefits to these patients, either by improving function or by preventing secondary complications [7]. Among the therapeutic approaches, pharmacological treatments, such as oral medications (e.g., tizanidine and dantrolene), intrathecal baclofen, as well as phenol or botulinum toxin injections, and non-pharmacological, such as electro-neuromuscular stimulation, orthotics, acupuncture, and physical activity programs have shown potential benefits [6,8]. Furthermore, surgical techniques such as nerve blockades, tendon lengthening, and selective neurectomy play a crucial role in reducing spasticity, correcting deformities, and improving limb functionality, especially when integrated within a multidisciplinary framework emphasizing comprehensive evaluation and dedicated post-surgery rehabilitation for the best outcomes [9,10,11].

The most widely accepted definition of spasticity is that of Lance et al., which includes this condition in UMNS: “Spasticity is a motor disorder characterized by a velocity-dependent increase in tonic stretch reflexes (muscle tone) with exaggerated tendon jerks, resulting from hyperexcitability of the stretch reflex, as one component of the upper motor neuron syndrome” [12]. This definition has evolved in clinical contexts to encompass a broader conceptualization of deforming spastic paresis, including not just spasticity but also related phenomena such as spastic dystonia and cocontraction, stretch-sensitive paresis, and soft tissue contracture [13]. In 2005, a new definition of spasticity was introduced by the Support Program for Assembly of a Database for Spasticity Measurement (SPASM). This definition characterizes spasticity as “disordered sensory-motor control, resulting from an upper motor neuron lesion”. [14]. As a sensory-motor phenomenon, it involves the sensory afferent and motor efferent systems, which suggests that it is not only a matter of terminology but also raises important neurobiological issues that should be examined in future research.

Sensory and motor alterations resulting from lesions in the central nervous system can impact interoception [15]. Interoception integrates signals from vital body systems (somatic, cardiovascular, gastrointestinal, and thermoregulatory) into conscious perception and regulation [16]. This complex process is central to physical, cognitive, and emotional self-management and significantly affects well-being [17,18]. Understanding and effectively interpreting these internal cues are essential for sustaining physical health, emotional stability, and cognitive function, highlighting the significant influence of interoception on human health and psychological resilience [19]. Following Craig’s theory, humans may experience a homeostatic emotion depending on the perception of a combination of a feeling and a motivation [20]. Behaviors driven by pleasant or unpleasant sensations that the body can perceive, such as temperature and pain, are referred to as homeostatically motivated behaviors [21]. In this view, the unpleasant sensations experienced by patients living with spasticity may affect their motivated behaviors, thereby affecting their overall quality of life [22]. Although the effect of stroke on body perception in stroke patients has been documented [23], the impact of unpleasant sensations related to spasticity on the functions and activities of individuals living with spasticity requires further investigation. In a study conducted by Baricich et al., individuals diagnosed with spastic paresis frequently encountered explicit bodily sensations, including pain, heaviness, and stiffness [24]. However, there is no evidence of the influence of unpleasant sensations on patients’ daily lives. Although the consequences of pain in individuals with UMNS have been widely examined in the literature [25,26], the same level of attention has not been paid to the experiences of heaviness and stiffness.

The International Classification of Functioning, Disability, and Health (ICF) is a universally accepted framework for classifying health and health-related domains [27]. The World Health Organization’s ICF model for health and disability employs a comprehensive biopsychosocial approach to document functional status and disability [27]. This approach involves Functioning and Disability, which includes Body Functions and Structures, Activities and Participation, and Contextual Factors, which encompass Environmental and Personal Factors [27]. Body function and structure refer to the anatomical parts and physiological functions of the body, including psychological functions [27]. This is where interoception is classified. Activities and Participation involve executing tasks and engaging with real-life situations. Contextual Factors interact with other domains and can either facilitate or hinder their functioning.

Employing the ICF framework, this study aimed to assess the relationship between unpleasant sensations related to spasticity and ICF domains, enriching our understanding of the impact of spasticity.

## 2. Materials and Methods

This study performed a secondary analysis of data previously collected in our investigations on the impact of Botulinum toxin type-A (BoNT-A) treatment discontinuation among individuals with stroke and TBI during the COVID-19 pandemic.

The study population included individuals aged ≥ 18 years who were experiencing spasticity as a result of stroke or TBI and receiving treatment with BoNT-A for more than one year. We developed an ad-hoc questionnaire to investigate the most important issues in patients living with spastic paresis covering the most important ICF domain. It was constructed utilizing an ICF framework, integrating insights from previous research on rehabilitation needs for spasticity and various existing assessment tools (e.g., Disability Assessment Scale, Numerical Rating Scale, Barthel Index, Short Form 36, ICF checklist [28,29,30,31,32]), and the authors’ patient-centered evaluation experience in spasticity treatment. In our study, we utilized an 11-point numerical rating scale (0–10) to assess the intensity of unpleasant sensations associated with spasticity and a 5-point Likert Scale (0–4) to quantitatively measure the degree of worsening during discontinuation of BoNT-A, noting that higher scores indicate worse outcomes. We explored items related to specific ICF domains: unpleasant sensations for the body function domain, mobility and self-care for the activities and participation domain, and facilitators for the environmental factors domain. Additionally, we investigated psychosocial aspects (mood, relationship, social life, motivation, and sleep quality) and assistance needs.

We specifically analyzed the Unpleasant Sensation subscore—aggregating pain, stiffness, and heaviness ratings for both limbs (ranging from 0 to 60)—to examine its distribution among participants. The higher the score, the higher the degree of worsening due to the discontinuation of BoNT-A treatment.

The full details of the methodology and the complete questionnaire have already been published in a study by Santamato et al. [33].

### Analysis of Data

Data were gathered from our recent research on the analysis of the need for spastic patients during forced discontinuation of BoNT-A treatment due to the COVID-19 pandemic [33]. The collected data were coded and analyzed using the Statistical Package for Social Science (SPSS 26, Armonk, NY, USA). Descriptive statistics were used to estimate the frequency and percentage of all variables. Quantitative variables are reported as mean ± standard deviation. Ordinal variables are reported as medians. Visual and statistical methods were used to evaluate the distribution of each variable. Histograms and quantile-quantile (Q-Q) plots were generated for each variable to visually assess the adherence to a normal distribution. For a formal assessment of normality, the Shapiro-Wilk test was conducted. Given the non-normal distribution of the data, the relationships between different variables were examined using Spearman rho correlation. The effect size was considered small (r = 0.1), medium (r = 0.3), or large (r = 0.5), according to Cohen [34]. For the purposes of this study, we considered as significant only correlations that had *p*-values < 0.001 and r > 0.2. We categorized the Unpleasant sensations by severity into four stratified groups: ‘None’ for a score of 0, ‘Mild’ for scores ranging from 1 to 3, ‘Moderate’ for scores between 4 and 6, and ‘Severe’ for scores from 7 to 10 [35,36]. To assess the differences in the intensity of unpleasant sensations between patients affected by TBI, ischemic stroke, and hemorrhagic stroke, we employed the Kruskal-Wallis test. For visual representation, data visualization was performed using Python (version 3.12) extensive libraries, including Matplotlib for creating charts and graphs, and Seaborn for generating informative statistical graphics. Scatter plots were generated for each psychosocial aspect against unpleasant sensations score which ranged from 0 to 60. These plots include linear regression trend lines to visually depict the correlation trends.

## 3. Results

The study comprised 151 participants, with a mean age of 58.42 ± 14.64 years. The majority of participants were male (59.6%, *n* = 90). The average time since the event was 7.81 ± 7.34 years. The characteristics of the population are detailed in Table 1.

The vast majority of participants (81.5%) were affected by stroke (75 ischemic, 48 hemorrhagic). The remaining 18.5% of the participants were affected by TBI. In the population examined, no statistically significant differences were observed in the score of unpleasant sensations among individuals with TBI, ischemic and hemorrhagic stroke (χ^2^(2) = 4.35, *p* = 0.113).

The most relevant percentages in Table 2 indicate that severe stiffness in the upper limb was reported by 50.3% of patients, whereas severe heaviness in the upper limb was experienced by 45% of patients. Additionally, severe stiffness in the lower limb was reported in 34.4% of the patients. Table 2 highlights that stiffness, especially in the upper limb, was the most prominent severe sensation experienced by our population. Within the observed patient group, 72.19% of the patients reported spastic pain in the upper or lower limbs, 98.01% experienced stiffness and 93.38% reported heaviness in either upper or lower limbs.

Most participants (75.76%) reported pain in the upper and/or lower limb. Most individuals experienced pain during movement, with 28.45% reporting pain exclusively during active movement and 15.52% during passive movement only. In addition, 10.3% of the participants felt pain during both passive and active movements. A substantial proportion (35.34%) of respondents experienced pain during both movement and rest, and 10.3% reported pain only at rest.

**Figure 1:**
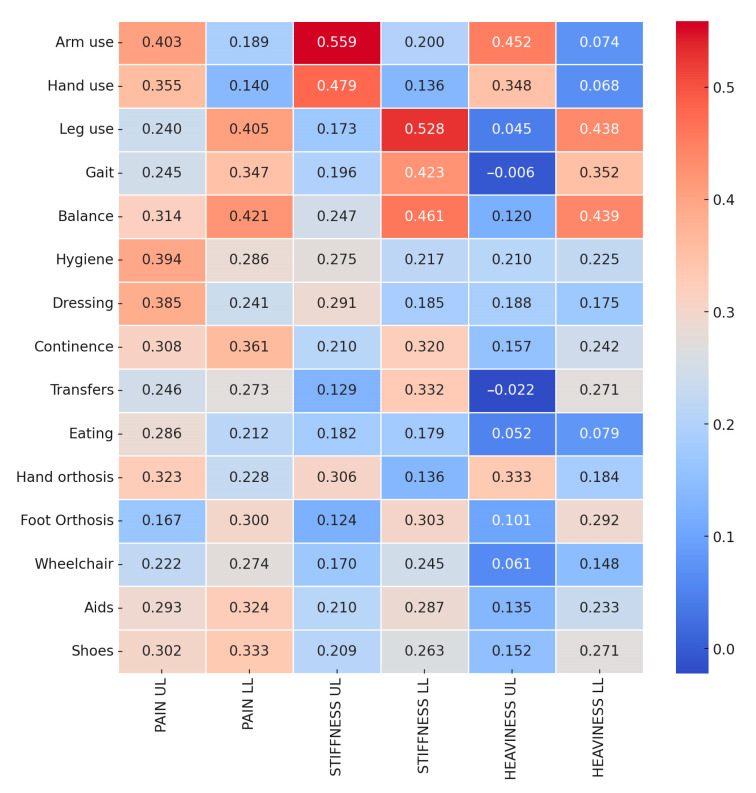
Correlation heatmap between Unpleasant sensations and activity domains of ICF.

We found a significant relationship between upper limb unpleasant sensations and upper limb functions and between lower limb sensations and lower limb functions (Figure 1). Self-care domains, including hygiene (d510), dressing (d540), eating (d550), and continence (d530), were significantly associated with all unpleasant sensations. Within the environmental factor domain (e115 and e120), 29.8% used foot orthosis, 32.45% used hand orthosis, 30.46% used wheelchairs, and 38.41% used aids. Additionally, the use of hand orthosis had a better correlation with upper limb unpleasant sensations, and the lower limb had a better correlation with the use of foot orthosis.

**Figure 2:**
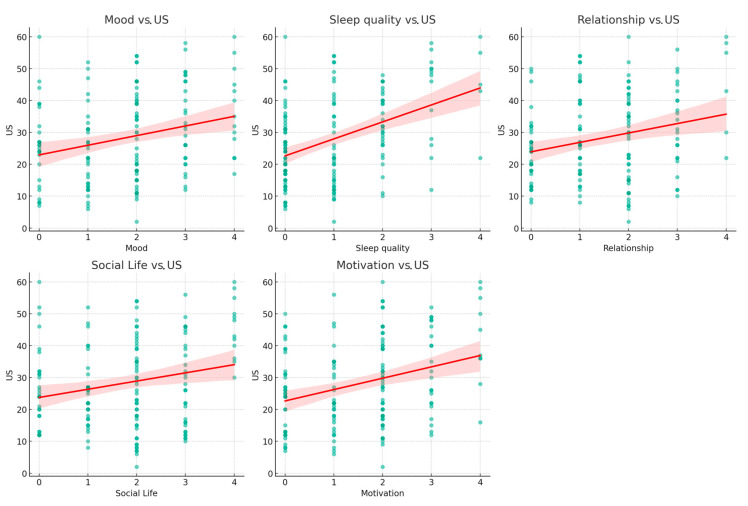
Scatter plots illustrating the correlations between Unpleasant Sensations and Psychosocial domains. Each subplot represents a bivariate scatter plot with individual observations (*n* = number not specified) demonstrating the relationship between self-reported psychosocial domains score (Mood, Sleep Quality, Relationship, Social Life, and Motivation, on a scale from 0 to 4) and US score (on a scale up to 60). The green dots on each scatter plot represent individual data points. The red lines signify the linear regression fits, suggesting a positive correlation, where increases in psychosocial domain scores were associated with higher US scores. The accompanying shaded regions delineate the 95% confidence intervals, providing insight into the precision of the regression estimates. US: Unpleasant sensations score.

We found a significant weak-to-moderate correlation between unpleasant sensations and psychosocial aspects (mood, sleep quality, relationships, and social life) that worsened during quarantine. Indeed, the participants reported worsening in all psychosocial aspects by increasing the overall Unpleasant sensations score (US) (Figure 2).

The Spearman correlation coefficient between ‘Mood’ and ‘US’ was found to be r = 0.260, indicating a positive correlation (*p* = 0.0093). A stronger positive correlation was noted between ‘Sleep quality’ and ‘US’, with an r = 0.461, which was highly significant (*p* < 0.001). Similarly, the correlation between ‘Relationship’ and ‘US’ was modest but significant (r = 0.295, *p* = 0.0030). The association between ‘Social Life’ and ‘US’ also showed a modest positive correlation (r = 0.276, *p* = 0.0056). Lastly, ‘Motivation’ was positively correlated with ‘US’ (r = 0.324, *p* = 0.0011).

## 4. Discussion

This study aimed to deepen the understanding of the relationship between unpleasant sensations caused by spasticity and the various domains outlined in ICF. In the context of spasticity-related sensations and their assessment, ICF is an expansive framework for the assessment of spasticity, encompassing more than mere clinical diagnosis and focusing on individuals’ functional capacities and their interaction with the environment [37]. This framework aids in understanding the link between interoceptive sensory processing and its effects on daily activities, participation, and overall quality of life [38]. Building on this foundation, our study aimed to explore the correlation between the severity of unpleasant sensations related to spasticity and self-reported limitations in daily activities and participation, as measured by an ad-hoc questionnaire meticulously developed to capture the extent of daily life restrictions that individuals might encounter due to their health condition or disability.

According to the data collected from our examined population, it was found that individuals suffering from spasticity post-stroke or TBI regularly experienced unpleasant sensations of pain, heaviness, and stiffness in their affected upper or lower limbs. These unpleasant sensations present significant challenges in definition and quantification due to their inherently subjective nature; given the pronounced interindividual differences, the only feasible method to measure these sensations is through subjective measures [39].

Measuring and reporting patient-centered endpoints, such as subjective sensations or opinions, may hold greater significance than gathering only objective clinical data [40]. Over the last few decades, the importance of patient experience has increased, and the movement towards personalized medicine has been significantly bolstered by the adoption of patient-reported outcome measures (PROMs), facilitating care tailored to the unique experiences and needs of individual patients [41,42]. Subjective pain is characterized by an individual’s personal experience of discomfort or distress, often described in terms of intensity, location, and quality, and can vary widely among individuals [43]. Stiffness refers to a subjective sensation of “rigidity” or “loss of flexibility” within a joint or muscle group, which may not directly correlate with biomechanical measures of resistance to movement, as with clinical measures (e.g., MAS or Tardieu Scale). This unpleasant sensation is commonly studied in arthritis [44,45,46]. However, there is a lack of research on neurological diseases. Finally, heaviness is described as a feeling of increased effort required to move a body part, a sensation that is not necessarily linked to the actual mass of the limb but rather to neurological or perceptual factors affecting movement perception [47,48]. The sensation of heaviness is not solely generated by central signals from the brain but is significantly influenced by peripheral signals, especially those arising from muscle spindles [49]. Since the muscle spindles’ activity was diminished due to paralysis, the feedback they provided about the muscle’s force and effort was altered, contributing to a sense of heaviness [49]. Although the intensity of these sensations varied, it was noted that severe stiffness of the upper limbs was the most common condition in our population, followed by heaviness and pain.

Our findings revealed significant correlations between unpleasant sensations in the limbs and the functional capabilities of individuals, with self-care activities being notably affected by these sensations. The correlation between upper limb unpleasant sensations and activities of daily living has substantial implications for patient care. In fact, in each of these domains, hygiene, dressing, eating, and continence—Unpleasant sensations are not just symptoms but a considerable barrier to independence and quality of life. Pain in the upper limbs showed a stronger correlation than stiffness and heaviness with both hygiene and dressing, suggesting that the experience of pain can severely hamper one’s ability to maintain personal cleanliness and attire independently [50]. The act of dressing involves a complex range of motions and fine motor skills, and pain can disrupt this delicate coordination [51], leading to reliance on assistance from caregivers or adaptive devices. Therefore, effective pain management is central to rehabilitation efforts that aim not only to alleviate discomfort but also to restore functionality and independence in daily living tasks [52].

The correlation between all unpleasant sensations in the upper limb and the use of hand orthosis hints at the potential of unpleasant sensations to impede assistive device usage. Studies have confirmed that discomfort, including pain and increased spasticity, leads to discontinuation or non-adherence to the recommended upper limb orthosis usage, thus limiting the therapeutic benefits of the devices [53]. It could be speculated that the unpleasant sensations may discourage the use of orthotic device utilization, highlighting the need for patient-centered design considerations in orthotic development to mitigate these challenges. Similarly, the use of foot orthosis was correlated with lower limb pain, indicating a relationship between pain levels and the use of these aids for lower limb spasticity. On one hand, the use of foot orthoses may contribute to providing structural support, improving alignment, and mitigating factors that exacerbate spasticity-related discomfort potentially enhancing physical performance and individual’s ability to perform daily activities [54]. On the other hand, the presence of orthoses can introduce or exacerbate pain, posing a significant barrier to their consistent use. Pain induced by wearing orthoses, alongside pre-existing pain related to spasticity, can deter patients from utilizing these potentially beneficial aids, thus negating their advantages and significantly impacting ADLs. It is important to recognize the complexity of pain experiences in spasticity, which can stem from a variety of sources including, but not limited to, muscular tension, nerve damage, or circulation issues [55]. Future studies are encouraged to delve deeper into the mechanisms by which foot orthoses influence pain perceptions, including distinguishing between various pain types such as neuropathic versus nociceptive pain, to further refine treatment strategies for individuals with spasticity. This suggests the need for device innovation or adjunct therapies that address Unpleasant sensations to enhance the utilization and effectiveness of these aids.

A recent study highlighted that individuals with spasticity, especially those with lower-limb spasticity undergoing BoNT-A treatment, prioritize mobility enhancement, pain management, and contracture prevention, underscoring a significant focus on maintaining and improving mobility [56]. Our research showed a correlation between unpleasant sensations in the lower limbs and mobility, which highlights the potential impact of Unpleasant sensations on the mobility-related goals of individuals with spasticity. In particular, all unpleasant sensations in the lower limbs had a notable correlation with gait, and stiffness in the lower limbs was also notably correlated with transfers, suggesting that increased stiffness may lead to challenges in moving. Furthermore, the correlation between balance and lower-limb unpleasant sensations findings indicates that these sensations could affect postural stability. Interestingly, a correlation was found between upper limb pain and the mobility domain (e.g., balance), underscoring the impact of pain on postural control mechanisms and the intricate relationship between body sensations and balance control. Pain can cause presynaptic inhibition of muscle afferents, leading to altered central modulation, ultimately affecting balance [57,58]. Research indicates that interventions targeting the management of spasticity and pain relief in the upper limb, such as the use of BoNT-A [59], not only mitigate spastic pain but also improve pathological posture. This suggests a complex interaction between upper limb conditions and balance control mechanisms, in which effectively addressing pain and spasticity can lead to significant improvements in balance and stability.

Ongoing struggles with motor difficulties and potential limitations in activities can lead to feelings of sadness, frustration, or anxiety [60]. Relationships and social life can also be affected, as individuals may find it challenging to participate in social activities or feel self-conscious about their motor difficulties, leading to social withdrawal or altered interactions with others [60]. Moreover, interoception plays a critical role in social interactions [61]. Individuals with spasticity who exhibit altered interoceptive processing may experience changes in their emotional state, motivation, and overall well-being, which can ultimately affect their ability to engage in adaptive behaviors. This alteration in interoception could hinder meaningful engagement with the environment, affecting self-awareness and trust in one’s body to accurately interpret and regulate interoceptive sensations. Our findings align with these observations, indicating that prevalent sensations of stiffness, heaviness, and pain among participants significantly affect their psychosocial well-being, including sleep quality, mood, motivation, and social interactions. As the unpleasant sensations score increased, the participants reported worsening psychosocial factors (Figure 2). Specifically, a correlation was observed between unpleasant sensations score and sleep quality, with similar trends in mood and social life mood, which can be affected by the chronic nature of spastic paresis.

These findings underscore the broader psychosocial ramifications of sensory disruption in this population. Sensory overload represents the brain’s overwhelming response to excessive sensory input where sensory stimuli are perceived as less or overly intense than their actual strength [62]. This condition is particularly prevalent in individuals with acquired brain injuries, where the conventional processing of sensory information is disrupted [63]. Such disruptions lead to altered perceptions that significantly influence daily living and functional recovery [64,65]. The heightened sensory input associated with spasticity-related unpleasant sensations may overwhelm the individual’s sensory processing capabilities, leading to a state of sensory overload. This state, in turn, exacerbates difficulties in regulating sleep patterns, maintaining stable moods, and engaging in social interactions [66,67]. Future research should aim to elucidate the causal pathways that connect sensory hypersensitivity in the form of spasticity-related unpleasant sensations to psychosocial outcomes. To this end, neuroimaging and neurophysiological techniques can potentially be leveraged to explore the underlying brain-behavior relationships.

### Study Limitations

This study acknowledges several limitations that may impact the interpretation and generalizability of its findings. First, reliance on self-reported data could introduce response bias, as participants may not accurately recall or choose to selectively report their behaviors and experiences. Second, the cross-sectional design of the study precludes the inference of causal relationships between the observed variables. Third, the study’s focus on individuals with stroke and TBI receiving BoNT-A treatment for over a year may restrict the generalizability of our results to other populations experiencing spasticity. Fourth, the use of an ad-hoc questionnaire, developed specifically for this investigation, might introduce biases related to the subjective interpretation of questions by participants. Additionally, we employed the NRS to evaluate subjective pain in individuals with spasticity. While the NRS is effective for quantifying pain intensity, it may not fully capture the distinctions between different pain types. Future research should address these limitations by employing prospective study designs, broadening the participant base to include diverse conditions causing spasticity, and utilizing validated measurement tools to enhance the reliability and applicability of the findings.

## 5. Conclusions

In conclusion, our study highlights the complex interplay between unpleasant sensations associated with spasticity, functional impairment, and psychosocial well-being. By exploring these relationships, our study contributes to a deeper understanding of the complex dynamics between sensory overload and psychosocial well-being, highlighting the importance of addressing sensory processing issues in therapeutic interventions for individuals with Stroke or TBI. The findings underscore the need for a person-centered approach in clinical practice, emphasizing SMART (Specific, Measurable, Achievable, Realistic/Relevant, and Timed) goals and the use of Goal Attainment Scaling for individualized treatment strategies [68]. Although effective, BoNT-A therapy should be aligned more closely with patient-specific needs and goals, rather than predominantly clinician-oriented beliefs. Future research should continue to explore innovative strategies to mitigate these sensory impairments and their repercussions, aiming to improve the overall health outcomes and quality of life of individuals with spasticity.

## Figures and Tables

**Table 1:** Characteristics of the population. See [33] for further details.

Characteristics	*n* (151)
*Gender*	
Male, *n* (%)	90 (59.6)
Female, *n* (%)	61 (40.4)
*Age* (mean ± SD, years)	58.42 ± 14.64
*Time since event* (mean ± SD, years)	7.81 ± 7.34
*Time since first injection* (mean ± SD, years)	3.07 ± 1.03
*Disease*	
Ischemic stroke, *n* (%)	75 (49.7)
Hemorrhagic stroke, *n* (%)	48 (3.,8)
Traumatic brain injury, *n* (%)	16 (10.6)
*Paretic side*	
Left, *n* (%)	60 (39.7)
Right, *n* (%)	80 (53)
Both, *n* (%)	11 (7.3)
*Affected limb*	
upper limb, *n* (%)	21 (13.9)
lower limb, *n* (%)	16 (10.6)
Both, *n* (%)	114 (75.5)

*n*: number of participants, SD: standard deviation.

**Table 2:** Distribution of Unpleasant Sensations by Severity and Median Scores (all participants and only symptomatic participants).

	Unpleasant Sensations					
	Pain UL	Pain LL	Stiffness UL	Stiffness LL	Heaviness UL	Heaviness LL
	*N* (%)	*N* (%)	*N* (%)	*N* (%)	*N* (%)	*N* (%)
none	54 (35.8)	58 (38.4)	18 (11.9)	16 (10.6)	26 (17.2)	23 (15.2)
mild	21 (13.9)	22 (14.6)	12 (7.9)	30 (19.9)	23 (15.2)	27 (17.9)
moderate	33 (21.9)	31 (20.5)	45 (29.8)	53 (35.1)	34 (22.5)	54 (35.8)
severe	43 (28.5)	40 (26.5)	76 (50.3)	52 (34.4)	68 (45)	47 (31.1)
Median score (min–max)	4 (0–10) 6 (1–10)	3 (0–10) 6 (1–10)	7 (0–10) 7 (1–10)	6 (0–10) 6 (1–10)	6 (0–10) 7 (1–10)	5 (0–10) 6 (1–10)

UL: Upper limb; LL: Lower limb.

## Data Availability

The original contributions presented in the study are included in the article, further inquiries can be directed to the corresponding author/s.
